# Multi-population GWAS and enrichment analyses reveal novel genomic regions and promising candidate genes underlying bovine milk fatty acid composition

**DOI:** 10.1186/s12864-019-5573-9

**Published:** 2019-03-06

**Authors:** G. Gebreyesus, A. J. Buitenhuis, N. A. Poulsen, M. H. P. W. Visker, Q. Zhang, H. J. F. van Valenberg, D. Sun, H. Bovenhuis

**Affiliations:** 1 Center for Quantitative Genetics and Genomics, Department of Molecular Biology and Genetics, Aarhus University, Blichers Allé 20, P.O. Box 50, DK-8830 Tjele, Denmark; 2 Animal Breeding and Genomics, Wageningen University and Research, P.O. Box 338, 6700 AH Wageningen, the Netherlands; 3 Department of Food Science, Aarhus University, Blichers Allé 20, P.O. Box 50, DK-8830 Tjele, Denmark; 4 Laboratory of Animal Genetics, Breeding and Reproduction, Ministry of Agriculture of China, National Engineering Laboratory for Animal Breeding, College of Animal Science and Technology, China Agricultural University, Beijing, 100193 China; 5 Dairy Science and Technology Group, Wageningen University and Research, P.O. Box 17, 6700 AA Wageningen, the Netherlands

**Keywords:** Milk fatty acids, Multi-population GWAS, Candidate genes, Pathway analysis

## Abstract

**Background:**

The power of genome-wide association studies (GWAS) is often limited by the sample size available for the analysis. Milk fatty acid (FA) traits are scarcely recorded due to expensive and time-consuming analytical techniques. Combining multi-population datasets can enhance the power of GWAS enabling detection of genomic region explaining medium to low proportions of the genetic variation. GWAS often detect broader genomic regions containing several positional candidate genes making it difficult to untangle the causative candidates. Post-GWAS analyses with data on pathways, ontology and tissue-specific gene expression status might allow prioritization among positional candidate genes.

**Results:**

Multi-population GWAS for 16 FA traits quantified using gas chromatography (GC) in sample populations of the Chinese, Danish and Dutch Holstein with high-density (HD) genotypes detects 56 genomic regions significantly associated to at least one of the studied FAs; some of which have not been previously reported. Pathways and gene ontology (GO) analyses suggest promising candidate genes on the novel regions including *OSBPL6* and *AGPS* on *Bos taurus* autosome (BTA) 2, *PRLH* on BTA 3, *SLC51B* on BTA 10, *ABCG5/8* on BTA 11 and *ALG5* on BTA 12. Novel genes in previously known regions, such as *FABP4* on BTA 14, *APOA1/5/7* on BTA 15 and *MGST2* on BTA 17, are also linked to important FA metabolic processes.

**Conclusion:**

Integration of multi-population GWAS and enrichment analyses enabled detection of several novel genomic regions, explaining relatively smaller fractions of the genetic variation, and revealed highly likely candidate genes underlying the effects. Detection of such regions and candidate genes will be crucial in understanding the complex genetic control of FA metabolism. The findings can also be used to augment genomic prediction models with regions collectively capturing most of the genetic variation in the milk FA traits.

**Electronic supplementary material:**

The online version of this article (10.1186/s12864-019-5573-9) contains supplementary material, which is available to authorized users.

## Background

Several fatty acids (FAs) of varying carbon chain length (C_4_-C_22_) and degree of saturation are present in milk. FAs in milk can originate either through direct transport from the rumen to the mammary gland via the blood, or from de novo synthesis in the mammary gland from acetate, beta-hydroxybutyrate [1] and propionate [2, 3]. Additionally, FAs in the mammary gland can originate from mobilization of body fat reserves. The short and intermediate chain FAs are mostly synthesized de novo in the mammary gland with the exception of C16:0, of which approximately 50% is assumed to be synthesized de novo. The long chain FAs, and approximately 50% of C16:0, are suggested to be derived from blood lipids originating from the diet [4] and mobilization of body fat reserves [1]. Considerable genetic variation has been reported for the fat composition of milk [5, 6]. Part of this genetic variation is attributed to polymorphisms in genes with major effects such as *DGAT1* and *SCD1* [7]. In addition, several regions on the bovine genome with suggestive effects on milk fat composition have been reported from GWAS [8–10]. Identified genes and genomic regions explain a fraction of 3.6 to 53% of the total genetic variation in different milk FA traits [8, 11]. Detection of additional genomic regions requires availability of larger sample size and high-density markers. GC analysis, the current method of choice to quantify milk FA, requires expensive equipment and is time-consuming, thus limiting measurement of the traits to experimental scale.

GWAS for the milk FA traits so far relied on such smaller datasets within different dairy cattle breeds/populations. An option to deal with the limitation in sample size could be to combine the available smaller datasets across populations for joint GWAS. Such analyses can increase detection power depending on the genetic distance between the populations and the marker density [12]. In this study, we undertake multi-population GWAS for milk FA traits by combining samples from Chinese, Danish and Dutch Holstein Friesians with HD genotypes available. Previous studies show high consistency in the linkage disequilibrium (LD) and minor allele frequencies between the populations [13, 14]. Thus, combining samples from these populations for joint GWAS might allow identification of genomic regions explaining even small proportions of the genetic variation in milk FA traits.

A hurdle is that due to the long range of LD in livestock breeds, GWAS often result in detection of large genomic regions [15] containing several positional candidate genes. Identifying the actual causative variants, therefore, requires additional evidence on top of the GWAS. Enrichment analysis is commonly undertaken in GWAS to prioritize positional candidate genes linked to significantly enriched pathways and gene ontology (GO) terms that are believed to be relevant to traits of interest. However, FA synthesis can take place in various mammalian tissues and thus further evidence is needed to determine whether such prioritized genes are relevant particularly to milk FA related mechanisms. Studies have been profiling differential expression of genes in the mammary tissues in various species [16, 17]. Information on expression status of genes in the mammary tissues can been used to further prioritize candidate genes linked to FA related pathways. Furthermore, the mammalian phenotype ontology [18], which provides annotation of mammalian phenotypes in the context of mutations, is increasingly becoming useful in fine-tuning the link between detected genes and phenotypes associated [19].

In this study, we implement GWAS for milk FA composition using multi-population dataset. Furthermore, we undertake post-GWAS analyses to identify, prioritize and functionally annotate genes within detected genomic regions using multiple information sources including Gene Ontology (GO), Kyoto Encyclopedia of Genes and Genomes (KEGG) pathways, mammary gland gene expression status and information in the mammalian phenotype ontology database [18].

## Results

### Descriptive statistics and genetic parameters

Table 1 presents phenotypic means, additive genetic variances and heritability estimates of the FAs expressed as weight percentage of total fat and the desaturation indexes in the combined multi-population dataset. The 13 FAs studied together amounted to 87.6% of total fat. Of the studied FAs, C18:3n3 and CLA occurred at concentrations less than 1% of total fat in the milk samples. Other FAs including C15:0, C8:0, C14:1 and C16:1 also occurred at low concentrations of total fat (means = 1.09–1.49). Coefficients of variation (not shown) of the FA traits ranged between 0.06% (C18 index) and 0.43% (CLA). Heritability estimates in the studied FA traits ranged from low (0.18) for C18:2n6 to high (0.53) for C14 index. The dataset used in the current study comprises samples from the Chinese, Danish and Dutch Holstein population and details regarding descriptive statistics and genetic parameters within each population can be found in our previous study (Submitted).

**Table 1:** Phenotypic means (with standard deviations, SD) and genetic parameters (with standard errors, SE) in the combined-population dataset

FAs	Mean	SD	${\sigma}_a^2$	SE	h^2^	SE
Saturated FAs^a^						
C8:0	1.18	0.38	0.008	0.04	0.27	0.03
C10:0	2.80	0.58	0.07	0.09	0.39	0.04
C12:0	3.58	0.76	0.09	0.11	0.33	0.04
C14:0	11.0	1.26	0.21	0.17	0.25	0.03
C15:0	1.09	0.18	0.004	0.02	0.23	0.04
C16:0	30.20	3.53	1.80	0.48	0.34	0.04
C18:0	10.30	1.99	0.52	0.29	0.25	0.04
Unsaturated FAs^a^						
C14:1	1.19	0.35	0.03	0.05	0.47	0.04
C16:1	1.49	0.35	0.05	0.07	0.46	0.04
C18:1c9	21.90	4.37	1.38	0.46	0.27	0.04
C18:2n6	1.89	1.19	0.01	0.05	0.18	0.03
C18:3n3	0.48	0.13	0.005	0.01	0.19	0.03
CLA	0.53	0.23	0.004	0.02	0.21	0.04
Desaturation indexes^b^						
C14 index	9.71	2.37	1.57	0.37	0.53	0.03
C16 index	4.70	0.97	0.32	0.19	0.38	0.04
C18 index	67.80	3.98	3.95	0.73	0.31	0.04

^a^Expressed in % wt/wt

^b^Desaturation indexes calculated as unsaturated/(unsaturated + saturated) × 100

### Detected genomic regions

Our multi-population GWAS resulted in the detection of 56 genomic regions containing single nucleotide polymorphisms (SNPs) significantly associated with at least one of the studied FA traits (Table 2). Significant associations were detected on all chromosomes except BTA 18. Most of the FA traits showed significant associations with multiple genomic regions on several chromosomes; particularly for C10:0 (14 regions), C16:0 (12 regions), C16:1 (13 regions), C18:1c9 (11 regions) and C16 index (13 regions). Proportions of genetic variance explained by the lead SNPs in the detected regions ranged between 1.4 and 45.3% for the different FA traits studied.

**Table 2:** Genomic regions associated with milk fatty acid traits in the multi-population analysis and suggested candidate genes

Region^a^	Start (Mbp)	End (Mbp)	Traits associated (and % of explained genetic variance)	Candidate genes
1a	19.92	19.93	C16:0_(3.1)_	
1b	101.0	101.0	C18 index_(2.8)_	
1c	141.3	142.5	C15:0_(3.9)_	
2a	12.5	19.8	C8:0_(3.7)_, C10:0_(3.0)_	*OSBPL6, AGPS*
2b	54.9	59.8	C14:1_(1.6)_, C16:0_(3.6)_, C16:1_(2.1)_, C14 index_(1.5)_	
2c	64.1	67.8	C16:1_(2.3)_, C16 index_(2.3)_	
2d	106.5	135.6	C12:0_(2.5)_, C15:0_(5.6)_, C16:0_(2.8)_, C18:1c9_(3.8)_	*MOGAT1, FABP3, MECR*
3	116.2	119.4	C18:3n3_(4.3),_ CLA_(3.2)_	*PRLH*
4	15.59	15.6	C15:0_(5.2)_	
5a	10.33	10.36	C15:0_(9.0)_	
5b	65.7	82.8	C8:0_(3.9)_, C10:0_(2.5)_	*CHPT1*
5c	87.4	99.0	C8:0_(4.3)_, C10:0_(3.2)_, C12:0_(2.6)_, C14:1_(1.7)_, C16:0_(2.7)_, C16:1_(2.1)_, C18:1c9_(5.6)_, CLA_(3.2)_, C14 index_(2.4)_, C16 index_(4.9)_	*MGST1, PLBD1, LRP6*
6	41.4	41.4	C18 index_(2.9)_	
7a	14.6	15.5	C8:0_(3.3)_, C10:0_(2.2)_	
7b	78.4	78.4	C18:2n6_(3.3)_	
7c	81.6	83.2	C12:0_(3.0)_, C15:0_(6.0)_	
8a	57.5	59.7	C15:0_(6.1)_, C16:1_(2.0)_, C16 index_(2.5)_	*PIGO, STOML2*
8b	79.9	98.4	C14:0_(3.9)_, C18:0_(4.1)_, CLA_(3.3)_	
9a	25.5	25.6	C14:1_(1.7)_	
9b	81.3	81.3	C15:0_(5.0)_	
10a	1.1	8.6	C10:0_(2.0)_, C12:0_(3.5)_	
10b	12.9	12.9	C14:1_(1.6)_, C18:0_(3.6)_	*SLC51B*
10c	78.1	80.1	C18:3n3_(4.9)_	*PIGH*
10d	87.5	93.1	C18:0_(4.1)_, CLA_(3.4)_, C18 index_(2.5)_	
11a	24.7	26.7	C16:0_(2.6)_	*ABCG5, ABCG8*
11b	58.81	58.89	C16:0_(2.8)_	
12a	17.1	17.1	C18:1c9_(3.5)_	
12b	24.0	24.8	C14:1_(1.8)_	*ALG5*
12c	70.0	77.4	CLA_(3.5)_, C16 index_(2.5)_	
13	64.6	65.7	C10:0_(2.4)_	*ACSS2*
14a	1.5	5	C8:0_(7.8)_, C10:0_(3.6)_, C14:0_(8.8)_, C14:1_(2.1)_, C15:0_(16.3)_, C16:0_(33.8)_, C16:1_(7.8)_, C18:1c9_(34.1)_, C18:2n6_(34.3)_, C18:3n3_(24.2)_, CLA_(14.6)_, C14 index_(4.5)_, C16 index_(11.3)_, C18 index_(11.4)_	*DGAT1, GPAA1*
14b	5.2	20	C8:0_(4.3)_, C10:0_(2.7)_, C15:0_(5.2)_, C16:0_(11.2)_, C16:1_(6.6)_, C18:1c9_(10.5)_, C18:2n6_(15.2)_, C18:3n3_(12.8)_, CLA_(4.7)_, C14 index_(1.8)_, C16 index_(3.4)_, C18 index_(4.4)_	*ST3GAL1*
14c	44.7	49.9	C14:1_(2.0)_, C16:1_(1.9)_, C14 index_(1.6),_ C18 index_(2.7)_	*PMP2, FABP9, FABP4* *FABP12*
15a	27.2	31.2	C10:0_(2.3)_, C14:0_(4.6)_, C18:0_(4.6)_	*APOA1, APOA4, APOA5, DPAGT1*
15b	46.9	65.9	C10:0_(2.8)_	*CAT*
16a	23.8	25.22	C18:0_(3.8)_, C16 index_(2.3)_	
16b	57.53	57.58	C16:1_(1.7)_, C16 index_(2.1)_	
17a	17.4	22.6	C16:1_(3.0)_, C16 index_(2.1)_	*MGST2*
17b	27.8	44.1	C8:0_(5.9)_, C10:0_(3.0)_, C16:1_(2.6)_, C18:3n3_(4.8)_, C16 index_(2.3)_	*LARP1B*
19	37.3	61.3	C8:0_(7.6)_, C10:0_(12.6)_, C12:0_(13.6)_, C14:0_(22.3)_, C16:0_(4.6)_, C18:1c9_(3.9)_, C14 index_(3.1),_ C18 index_(2.5)_	*ACLY, BRCA1, FASN, STAT5A,*
20a	32.4	34.2	C16:1_(1.9)_, C18:0_(4.3)_	*PRKAA1*
20b	36.7	36.9	C14:1_(1.6)_, C18:1c9_(3.9)_	
20c	55.3	60.4	C14 index_(1.6)_, C18 index_(2.8)_	
21	53.8	59.1	C10:0_(2.3)_, C12:0_(2.9)_, C14:0_(3.3)_, C18:1c9_(4.1)_	
22	59.12	59.13	C14 index_(1.6)_	
23a	26.7	32.7	CLA_(4.3)_	*AGPAT1, ATAT1*
23b	33.5	36.5	C15:0_(5.8)_	
23c	40.7	43.5	C18:1c9_(3.4)_, C16 index_(2.1)_, C18 index_(2.6)_	
24	10.2	10.2	C18:0_(4.2)_	
25a	9.8	9.9	C12:0_(3.1)_	
25b	24.7	24.7	C18:1c9_(3.5)_	
25c	41.4	41.7	CLA_(3.0)_ C14 index_(1.4)_	
26	2.9	43.0	C8:0_(3.7)_, C10:0_(5.5)_, C12:0_(3.3)_, C14:0_(8.0)_, C14:1_(39.0)_, C16:0_(2.4),_ C16:1_(13.6)_, C18:0_(4.5)_, C14 index_(45.3)_, C16 index_(19.7)_, C18 index_(3.3)_	*SCD, ELOVL3, ACSL5, GPAM*
27	37.0	42.2	C16:0_(2.9)_	
28	36.6	37.2	C16:1_(2.3)_, C16 index_(2.5)_	
29	32.9	40.5	C16:0_(2.5)_, C18:1c9_(3.2)_	*TKFC*

^a^BTA number with subscript of alphabets to denote the multiple regions within a chromosome

**Fig. 1:**
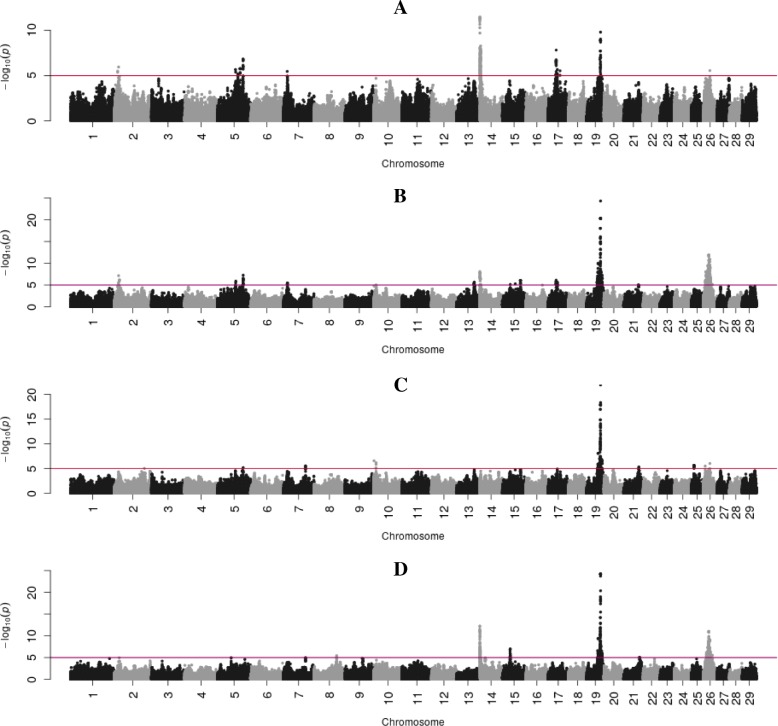
Manhattan plots showing BTAs on the x-axis and -log 10-*p* values on the y-axis for the de novo synthesized FAs of C8:0 (**a**), C10:0 (**b**), C12:0 (**c**), C14:0(**d**). Red line indicates the significance threshold (log 10 *p*-value =5.0)

**Fig. 2:**
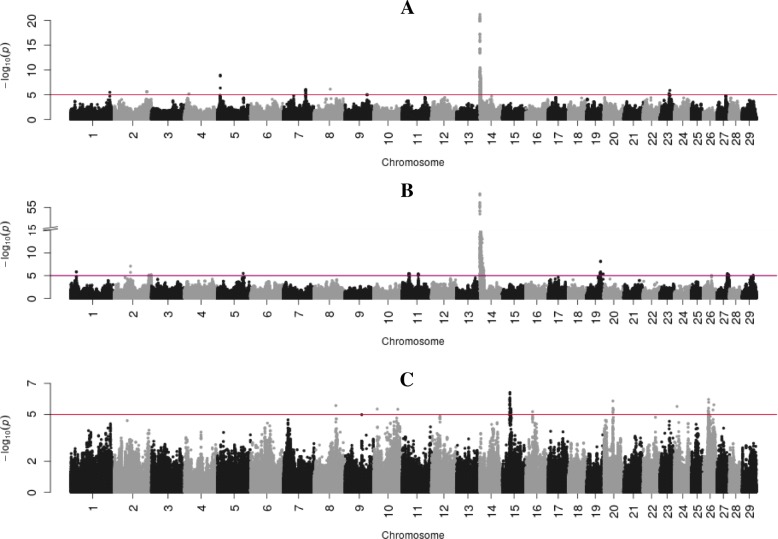
Manhattan plots with BTAs on the x-axis and -log 10-*p* values on the y-axis for the medium to long-chain FAs of C15:0 (**a**), C16:0 (**b**), C18:0 (**c**). y-axis for (**b**) has breaks at –log 10-*p*-value =15 to show only the highest values of those –log 10-*p*-value > 25 while keeping the visibility of smaller peaks. Red line indicates the significance threshold (log 10 *p*-value =5.0)

**Fig. 3:**
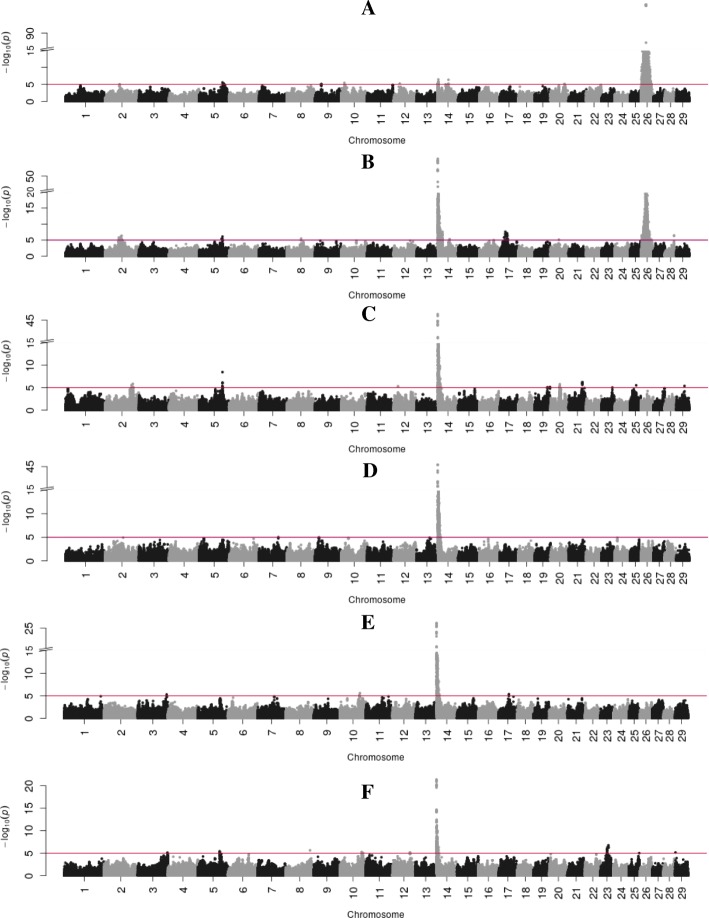
Manhattan plots showing BTAs on the x-axis and -log 10-*p* values on the y-axis for the unsaturated FAs of C14:1 (**a**), C16:1 (**b**), C18:1c9 (**c**), C18_2n6 (**d**), C18_3n3 (**e**), CLA (**f**). Y-axis breaks for (**b**) at –log 10-*p*-value =20 and for (**a**), (**c**) and (**d**) at –log 10-*p*-value = 15. Red line indicates the significance threshold (log 10 *p*-value =5.0)

**Fig. 4:**
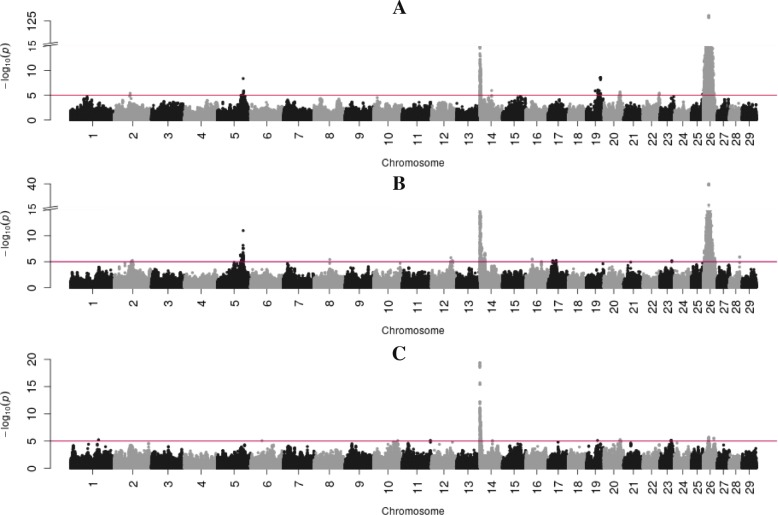
Manhattan plots showing BTAs on the x-axis and -log 10-*p* values on the y-axis for the desaturation indexes: C14 index (**a**), C16 index (**b**), C18 index (**c**) with y-axis breaks at –log 10 *p*-values = 15 for (**a**) and (**b**). Red line indicates the significance threshold (log 10 *p*-value =5.0)

Peak sizes (highest –log 10 *p*-value) across FA traits ranged from a –log 10 *p*-value of 6.9 for C18:0 to a –log 10 *p*-value of 126 for C14 index. Figs. 1, 2, 3 and 4 present Manhattan plots for all FAs according to the different FA groups i.e., de novo FAs (Fig. 1), intermediate to long-chain saturated FAs (Fig. 2), the unsaturated FAs (Fig. 3), and desaturation indexes (Fig. 4). The strongest association for C8:0 (−log10 *p*-value = 11.39), C15:0 (−log10 *p*-value = 21), C16:0 (−log10 *p*-value = 58), C16:1 (−log10 *p*-value = 55), C18:1c9 (−log10 *p*-value = 46), C18:2n6 (−log10 *p*-value = 29), C18:3n3 (−log10 *p*-value = 24.8), CLA (−log10 *p*-value = 18.1) and C18 index (−log10 *p*-value = 19.3) was observed at two variants on BTA 14 (ARS-BFGL-NGS-4939 and BovineHD1400000216). This region (14a) was significantly associated with all studied FA traits except C12:0. The lead SNP in this region explained up to 34% of the genetic variation in C18:1c9 and C18:2n6. Two other regions on BTA 14 remained significantly associated with multiple FA traits after accounting for the fixed effect of the lead SNP from region 14a (ARS-BFGL-NGS-4939). The second region (14b) was also significantly associated with most FA traits except C12:0. The third region on BTA 14 (14c), was significantly associated with C14:1, C16:1, C14 index and C18 index. The lead SNP in this region explained 2.7% of the genetic variation in C18 index and 1.6% in C14 index.

Strongest association for C10:0 (−log10 *p*-value = 24.3), C12:0 (−log10 *p*-value = 22) and C14:0 (24.2) was detected with two variants on BTA 19 (BovineHD1900014372 and BovineHD1900014348). Significant associations were also detected for C8:0, C16:0, C18:1c9, C14 index and C18 index with SNPs located between 37.3 to 61.3 Mbp on chromosome 19. Particularly for C14:0, 22.3% of the genetic variation was explained by the lead SNP in this region.

The strongest association for C14:1 (−log10 *p*-value = 98.8), C14 index (−log10 *p*-value = 126) and C16 index (−log10 *p*-value = 39.8) was found with SNPs on chromosome 26 (BovineHD2600005461). Significant associations were also detected for C8:0, C10:0, C12:0, C14:0, C16:0, C16:1, C18:0 and C18 index. The lead SNP in this region explained 39.0% of the genetic variation in C14:1.

Effects of lead SNPs in all the detected genomic regions are presented in Additional file 1. In general for most of the regions, directions of effects were opposite for the de novo synthesized FAs versus the long chain FAs.

### Gene assignment and functional annotations

Several genes positioned within the detected genomic regions were retrieved from the ensemble database. These positional candidate genes were further prioritized using enrichment analyses implemented in the DAVID web platform (https://david.ncifcrf.gov), which resulted in different significantly enriched GO terms and KEGG pathways relevant to FA related mechanisms (Table 3).

**Table 3:** The list of enriched FA related pathways and GO terms

Category	Term	Count	*P*_value	FDR
GOTERM_BP_DIRECT	GO:0006633~fatty acid biosynthetic process	8	< 0.001	< 0.001
GOTERM_MF_DIRECT	GO:0008289~lipid binding	13	< 0.001	< 0.001
GOTERM_BP_DIRECT	GO:0070328~triglyceride homeostasis	5	< 0.001	< 0.001
GOTERM_BP_DIRECT	GO:0008610~lipid biosynthetic process	13	< 0.001	0.002
GOTERM_BP_DIRECT	GO:0016042~lipid catabolic process	15	< 0.001	< 0.001
GOTERM_BP_DIRECT	GO:0045717~negative regulation of fatty acid biosynthetic process	4	< 0.001	0.001
GOTERM_BT_ALL	GO:0010876~lipid localization	9	0.001	0.01
GOTERM_MF_DIRECT	GO:0005543~phospholipid binding	5	0.001	0.02
GOTERM_BP_DIRECT	GO:0006631~fatty acid metabolic process	20	0.005	0.01
GOTERM_BP_DIRECT	GO:0046486~glycerolipid metabolic process	9	0.03	0.01
UP_KEYWORDS	Acyltransferase	10	< 0.001	< 0.001
UP_KEYWORDS	Lipid transport	7	< 0.001	< 0.001
INTERPRO	IPR016181:Acyl-CoA N-acyltransferase	6	< 0.001	< 0.001
KEGG_PATHWAY	bta00564:Glycerophospholipid metabolism	10	< 0.001	< 0.001
KEGG_PATHWAY	bta04975:Fat digestion and absorption	10	< 0.001	< 0.001
KEGG_PATHWAY	bta00565:Ether lipid metabolism	8	< 0.001	< 0.001
KEGG_PATHWAY	bta00062:Fatty acid elongation	4	0.004	0.04
KEGG_PATHWAY	bta05204:Chemical carcinogenesis	3	0.004	0.04

Among the enriched GO terms and pathways were biosynthesis related, such as ‘GO:0006633~FA biosynthetic process’, binding and transport related, such as ‘GO:0008289~lipid binding’ and ‘GO:0010876~lipid localization’, and metabolism, such as ‘GO:0006631~FA metabolic process’ and ‘bta00564:Glycerophospholipid metabolism’.

Some among the set of genes in all significantly enriched pathways and GO terms (Additional file 2) were also found to be expressed in mammary tissues and epithelial cells across different species. Furthermore, some of the prioritized candidate genes were linked to abnormalities related to FA metabolism in the mammalian phenotype database including ‘increased circulating triglyceride levels’ (MP:0001552), ‘abnormal lipid homeostasis’ (MP:0002118) and ‘abnormal phospholipid level’ (MP:0004777).

Apart from genes, also non-coding genomic features such as micro RNAs were located within the detected genomic regions as presented on Additional file 3.

## Discussion

### Agreement between detected regions and previous reports

Our multi-population GWAS resulted in detection of large numbers of genomic regions significantly associated with at least one of the 16 milk FA traits studied, indicating the complexity of the milk FA synthesis pathways. Most of the detected genomic regions have been previously reported in connection to milk FA traits, e.g. genomic regions on BTA 14, BTA 19 and BTA 26 [8, 10, 20].

On BTA 14, our analysis indicates three distinct regions significantly associated with several FA traits. The first region is known to contain the *DGAT1* gene, of which the effects are well established for multiple FA traits [21, 22]. The second region was previously reported to show significant associations with milk fat percentage [23]. The boundaries of these two regions (14a and 14b) are in close proximity of each other (1.5–5 Mbp and 5.2–20 Mbp) and the regions appear to be highly correlated in terms of associated FA traits and proportions of genetic variance explained for these traits. While our analysis indicates two distinctive regions, Bouwman et al. [8], based on part of the dataset used in our study, reported a single, broader region (0.0–26.3 Mbp) with significant associations to several FA traits. Our hypothesis is that different quantitative trait loci (QTL) underlie these two regions (14a and 14b) but that estimated effects of the QTLs could be confounded, because the high LD at the start of BTA 14 [24] makes it difficult to disentangle the effects of multiple QTL.

The third region on BTA 14 (44.7–49.9 Mbp) was exclusively associated with C14:1 and C16:1 as well as C14 index and C18 index. This region was previously reported for significant associations with C16:1 [8] and milk fat percentage [25]. The region contains the fatty acid binding proteins *FABP4*, *FABP9* and *FABP12* as well as the peripheral myelin protein (*PMP2*), enriching the GO terms of FA metabolic process (GO:0006631) and lipid binding activities (GO:0008289). A study by Nafikov et al. [26] reported a *FABP4* haplotype negatively associated with saturated milk FAs and the ratio between saturated and unsaturated FAs while having positive effects on the unsaturated FAs. Marchitelli et al. [27] also reported that the *FABP4* affected the ratio of monounsaturated/saturated FA in milk. Additionally, variation in *FABP4* is reported to affect other milk production traits such as milk yield [28]. Therefore, results of our analysis and previous studies suggest a role of this region in desaturation of C14:0, C16:0 and C18:0 with the *FABP4* as the most likely candidate gene.

Broader regions were detected on BTA 19 (37.3–61.3 Mbp) and BTA 26 (2.9–43.0 Mbp). The genes *FASN* on BTA 19 [29] and *SCD1* on BTA 26 [30] have previously been suggested as the likely candidate genes for FA traits. However, our enrichment analysis indicate additional genes in these regions connected to important FA metabolism processes including the *ACLY*, *STAT5a, PRKAA1, GH* on BTA 19 and *ELOVL3, ACLS5* on BTA 26. Significant associations were previously reported between variants within some of these genes and some milk FA traits [11, 31].

In our study, more FA traits have been found to have significant associations with the *DGAT1* and *SCD1* regions than previous GWAS using different parts of the multi-population dataset used in the current analysis [8–11, 14]. These previous studies might not be considered as independent of the current analysis; however, more associations in the current analysis can be an indication of improved detection power from combining the populations. This was also demonstrated in our previous study (Submitted) in which results of population-specific analyses versus multi-population joint GWAS were compared. Effects of the *DGAT1* (ARS-BFGL-NGS-4939) and *SCD1* (BovineHD2600005461) loci were similar in direction and highly correlated between the three populations but estimated effects in the Chinese sample were consistently lower across the FAs compared to the Dutch and Danish Holstein samples.

The three regions detected on BTA 5 overlap with previously reported regions for milk FA traits [8, 9, 32]. For region 5c, *MGST1* was suggested as the most likely candidate gene [32]. In our analysis, the lead SNP in the region was located within the *MGST1* gene. However, our enrichment analysis did not establish any connection to *MGST1* with significantly enriched FA related GO terms and pathways. Additionally, *PLBD1* and *LRP6* genes were connected to several pathways including lipid localization (GO:0010876) and transport (UP_KEYWORDS) suggesting that the significant association observed in the region with 10 FA traits might not be limited to the *MGST1* effect.

The region on BTA 13 was previously detected in the Dutch Holstein population [8, 11] and in Danish Jersey [9] with both studies suggesting the *ACSS2* as the highly likely candidate gene. Meanwhile, using infrared (IR) predicted phenotypes for the de novo FAs, Olsen et al. [33] suggested that the *NCOA6*, not the *ACSS2*, is responsible for significant associations in the region. Our enrichment analysis however links *ACSS2* with several significantly enriched pathways while no such links were established for the *NCOA6* gene.

Similarly, the first region on BTA 15 (27.2–31.2 Mbp) has been reported in previous studies including a joint Chinese-Danish Holstein population [14]. Several genes enriching FA related pathways were detected in the region including *APOA1, APOA4, APOA5,* and *DPAGT1*. The apolipoproteins *APOA1/4/5* enriched glycerolipid metabolic process (GO:0046486), fat digestion and absorption (bta04975) as well as negative regulation of FA biosynthetic process (GO:0045717) while the *DPGAT1* was involved in lipid biosynthetic process (GO:0008610). The strongest associations observed in the region were between C18.0 and variants within the alipoprotein genes, which showed opposite direction of effects on C10:0 and C14:0. Although effects were not significant, the lead SNP in the region also showed moderate effects on the other de novo FAs including C8:0 (−log 10 *p*-value = 2.96) and C12:0 (−log 10 *p*-value = 2.96) with direction of effects similar to C10:0 and C14:0. The alipoproteins APOA1/4/5 are thus collectively suggested as the candidates underlying the strong effect on C18:0 observed in the region. The opposing effects on the de novo FAs might be directly through involvement of the alipoproteins in negative regulation of FA biosynthesis or indirectly through the effect on C18:0, which suppresses de novo synthesis.

The two regions detected on BTA 17 are also in agreement with previous findings. The regions detected by Bouwman et al. (2012) [8] (15.0–23.9 Mbp) and Li et al., [10] (19.5–22.5 Mbp) overlap with the first region (17a) detected in our study. In the region, *MGST2* significantly enriched GO terms that included FA metabolic (GO:0006631) and biosynthetic (GO:0006633) processes. The *MGST2* is previously linked to intramuscular FA composition in pigs [34] and shown to be expressed in all stages of lactation in humans [17]. Therefore, the *MGST2* is suggested as the likely candidate gene underlying effects on the first region of BTA 17. Using a subset of the dataset used in the current study to fine map BTA 17, Duchemin et al. [35] suggested the *LARP1B* as a primary candidate gene in the second region (17b). However, our enrichment analysis did not result in significant enrichment of any of the FA pathways and ontology terms for genes in the region.

Some of the regions detected in our analysis overlap with results from some of the recently published GWAS that are based on IR predicted FA phenotypes [33, 36]. Interestingly, some of the well-established genomic regions in connection to GC-based FA traits, which were also detected in our analysis, have not been found to have significant associations with any of the milk FA phenotypes in these studies. For instance, GWAS by Olsen et al. [33] and Knutsen et al. [36] using the IR predicted FA phenotypes in Nordic Red cattle did not detect any significant association in the *DGAT1* and *SCD1* regions. Lack of segregation of the A variant of the DGAT1 K232A polymorphism has been suggested as the potential reason for the lack of association in the *DGAT1* region. Additionally, Wang et al. [37] showed that the *SCD1* polymorphism did not significantly affect any of the milk IR wavenumbers in samples from the Dutch Holstein population. These findings suggest that IR predicted FA phenotypes are not suitable for GWAS. While some FAs can be accurately predicted based on IR [38], low prediction accuracies [39] and low genetic correlations with GC measured FA [40] have been reported for other FAs. Especially FAs found in low concentrations in milk were shown to have low IR prediction accuracies [41]. Apparently, the power to detect QTL can be severely restricted by the IR prediction accuracy.

### Novel genomic regions and candidate genes

Of the 56 genomic regions significantly associated with at least one FA trait in this study, regions located on BTA 2, 3 10, 11, 12 and 21 appear to be novel regions that have not been previously connected to milk FA traits. The lead SNPs in these regions explained between 1.4 and 5% of the genetic variation in at least one of the FA traits studied.

### BTA 2

**Fig. 5:**
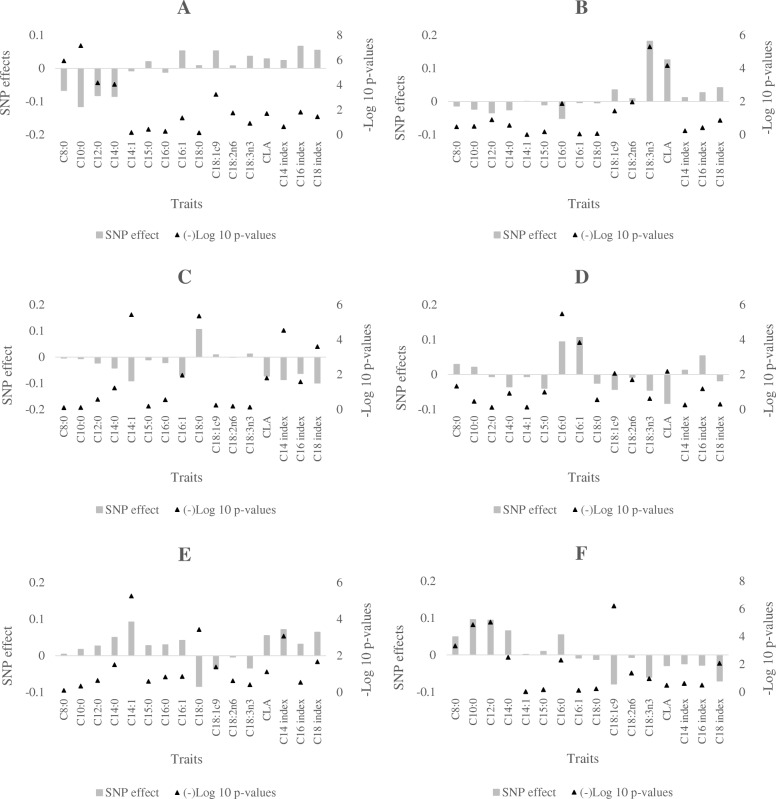
Effects of lead SNPs on regions 2a (**a**), 3 (**b**), 10b (**c**), 11b (**d**), 12b (**e**) and 21 (**f**) standardized by dividing the SNP effects with standard deviation of the respective FA trait

Two genes retrieved for region 2a enriched GO terms related to fatty acids. The *OSBPL6* gene belonging to the oxysterol-binding protein (OSBP) family, a group of intracellular lipid receptors, is shown to be involved in lipid binding (GO:0008289) and transport (UP_KEYWORDS) processes. The *OSBPL6* gene is found to be expressed in the human mammary gland during several stages of lactation [17]. The human *OSBPL6* gene is also shown to have a binding site for miR-33a/b [42], which is a microRNA shown to have targeting effects on genes regulating β-oxidation of FAs [43], leading to significantly lower levels of β-hydroxybutyrate [44]. Another gene located in the region (*AGPS*) also enriched GO terms related to FA synthesis including lipid biosynthesis process (GO:0008610). In the mammalian phenotype database, mutation in the *AGPS* gene in mice has been linked to abnormal lipid levels (MP:0001547), which is a rather broad term in the database referring to any anomaly in the concentrations of fat-soluble substances in the body, including circulating triglyceride and free FAs. Thus, our enrichment analysis indicate that both the *OSBPL6* and *AGPS* might have roles on de novo synthesis of FAs. Pattern of SNP effects in the region is also in agreement with enrichment analysis such that strongest association was estimated with C8:0 and C10:0 while moderate, but not significant effect was measured for C12:0 and C14:0 (−log 10 *p*-value = 4.2). Opposing direction of the lead SNP effect were also observed for the de novo synthesized FAs, except C15:0, on the one hand and most of the long chain FAs on the other (Fig. 5a). Therefore, both the *OSBPL6* and *AGPS* are considered as likely candidates in the region. .

### BTA 3

On the detected novel region of BTA 3, the prolactin releasing hormone (*PRLH)* was shown to be involved in FA metabolic process (GO:0006631). Mutations on the *PRLH* gene in mice have been associated with increased circulating triglyceride levels (MP:0001552) and increased total body fat amount (MP:0010024) in the mammalian phenotype database. In mammals, the *PRLH* gene is known to stimulate prolactin release and regulate its expression. Prolactin, which is a polypeptide hormone, has been shown to induce lipogenesis in many tissues [45] and stimulate the expression of genes involved in milk protein synthesis and lipid metabolism [46–48]. Moreover, prolactin has been shown to have a wide-range of effects on lactation including growth and development of the mammary gland, promotion of milk synthesis and maintenance of milk secretion [49–51]. Therefore, the *PRLH* gene*,* through regulation of prolactin release might have effects on milk yield. The pattern of SNP effects in the region suggest a connection with the poly-unsaturated fatty acids (PUFAs) with strongest associations observed for C18:3n3 and CLA. The direction of effects of the lead SNP was similar for all unsaturated FAs as well as all the desaturation indexes, while opposing effects were estimated for the de novo synthesized FAs and C16:0 (Fig. 5b). C16:0 is shown to have strong negative genetic correlation with milk yield, while moderate positive correlations were reported for the PUFAs [5]. Therefore, the *PRLH* is suggested as the candidate gene in the region; the effect of which might be indirect through its effect on milk yield, affecting the concentration of the PUFAs and C16:0.

### BTA 10

The second region on BTA 10 contains the solute carrier family 51-beta subunit (*SLC51B*) genewhich is implicated in lipid transport (UP_KEYWORDS) and localization (GO:0010876) processes. Pattern of effects in the region show strong effect on C14:1 and moderate effects on with C14 index (−log 10 *p*-value = 4.5) and C18 index (−log 10 *p*-value = 3.6) in direction opposite to the strong effect on C18:0 (Fig. 5c). This pattern suggests a reduction in desaturation when C18:0 increases. C18:0 in milk is largely derived via direct transport through the blood from the rumen where is it formed from bio-hydrogenation of dietary C18:2n6 and C18:3n3. Therefore, the effect of *SLC51B* is highly likely through its involvement in the FA transport processes. Dietary poly-unsaturated FAs, such as C18:2n6, are known to suppress SCD1 activity, thereby reducing its desaturation activity [52]. Thus, we hypothesize that *SLC51B* underlies the effect on C18:0, while observed opposite effects on the unsaturated FAs and desaturation indexes are rather due to the correlation in C18:0 in milk and dietary PUFA, which suppress desaturation.

### BTA 11

Among the genes located in the first region of BTA 11, the ATP binding cassette subfamily G5 (*ABCG5*) and *ABCG8* enriched several pathways and processes including fat digestion and absorption pathway (KEGG ~bta04975) and the GO terms of lipid localization (GO:0010876) and transport. In the mammalian phenotype database, the *ABCG5* and *ABCG8* genes are linked to increased circulating triglyceride level (MP:0001552), abnormal lipid homeostasis (MP:0002118) and abnormal phospholipid level (MP:0004777). In humans, mutations in *ABCG5/8* have been linked to conditions characterized by abnormal accumulation of sterols in blood and tissues [53, 54] implicating them in lipid absorption and transport. The KEGG pathway for fat digestion and absorption involves absorption of lipid from the rumen to the blood stream and from the blood stream to the mammary gland. Viturro et al. [55] previously reported high expression levels of both *ABCG5* and *ABCG8* genes in bovine liver, mammary gland, digestive tract and blood samples. Expression of ABCG5/8 in bovine mammary gland might indicate that apart from absorption and transport of lipids from the digestive tract, *ABCG5*/8 might also be involved in the secretion of lipids from the mammary gland into the milk. Significant association in the region was limited to C16:0. Although not significant, this region was also associated with C16:1 (−log 10-pvalue = 3.8) and CLA (−log 10-pvalue = 2.2), with directions of effects on CLA opposite to the effects on C16:0, C16:1, and C16 index (Fig. 5d). The GO term of lipid localization and association almost exclusively with C16:0, which is one of the FAs that is highly mobilized from body reserves during negative energy balance, might also indicate a role in the mechanism of body fat reserve mobilization.

### BTA 12

The dolichyl-phosphate beta glucosyltransferase (*ALG5*) gene located on the second region of BTA 12 was shown to enrich the lipid biosynthesis process (GO:0008610) and glycerolipid metabolic process (GO:0046486). The ALG5 gene has previously been shown to be differentially expressed during the different stages of lactation in bovine [16] and human [17]. Significant effects in the region were limited to C14:1. The lead SNP also showed moderate effect on C14 index (−log 10 *p*-value = 3.07) and C18:0 (−log 10 *p*-value = 3.43) where opposite direction of effects were observed for C18:0 (Fig. 5e). Therefore, the *ALG5* is suggested as promising candidate for further characterization for potential role in desaturation process.

### BTA 21

Significant associations were detected on BTA 21 with C10:0, C12:0, C14:0 and C18:1c9. Effects estimated for the lead SNP in the region were generally opposing directions for the de novo synthesized FAs and C16:0 versus for the long-chain FAs and desaturation indexes (Fig. 5f). Significant associations have previously been reported in the region with milk and milk protein yield [56] as well as fertility traits in cows [57]. However, our enrichment analysis show no gene implicated in the significantly enriched pathways and Go terms. Despite lack of genes implicated on FA related pathways, moderate effects observed for multiple traits in the region are of particular interest. QTL detected through GWAS might be located in non-coding regions. Such QTLs might be involved in regulation of expression of other genes affecting the traits of interest. Therefore, the region might be of interest for eQTL based GWAS in milk FA traits.

### Regulatory elements within detected genomic regions

Apart from coding genes, retrieved genes from the detected regions included regulatory elements, most commonly microRNAs (miRNAs). MiRNAs are small RNAs that regulate the expression of complementary messenger RNAs [58]. Several studies have reported possible roles of miRNAs in lipid and fatty acid metabolisms and in mammary gland development and lactation in several species [59–61]. Some of the miRNAs in the detected genomic regions in our study were previously linked to regulatory roles on genes related to FA metabolism and synthesis. Of these, bta-mir-27b, on BTA 8 (region 8b) was shown to target known FA synthesis genes such as *FASN* and *SCD1* [62] as well as mRNAs involved in lipid metabolism [63] and shown to be highly expressed during different stages of bovine lactation [64]. Among the genes located on BTA 2 (region 2d), the bta-mir-26b was shown to be expressed in bovine milk cells and mammary gland [60]. Wang et al., [61] showed that downregulation of miR-26a/b and their host genes decreased the expression of genes related to fatty acid synthesis, including *DGAT1* and *SCD1*.

## Conclusion

Multi-population GWAS for GC-quantified FA traits resulted in the detection of 56 genomic regions significantly associated to at least one of the studied FAs, including novel regions explaining relatively smaller fractions of the genetic variation. Enrichment analysis of genes harbored in detected regions reveals promising candidate genes some of which have not been previously linked to milk FA traits, including *OSBPL6* and *AGPS* on BTA 2, *PRLH* on BTA 3, *SLC51B* on BTA 10, *ABCG5/8* on BTA 11 and *ALG5* on BTA 12. Post-GWAS analyses using multiple data sources on pathways, ontology terms and tissue-specific gene expression status enabled prioritization of highly likely causative candidate genes among several positional candidates on detected regions. Use of such data in combination in combination with analysis of patterns of effects across the milk FA spectrum allowed linking some of the candidates to specific FA synthesis mechanisms. Detection of several novel regions and candidate genes will be contribute to the knowledge base on genetics underlying the bovine milk FA composition.

## Methods

### Animals and phenotypes

Test-day milk samples were obtained from 784 Chinese, 675 Danish and 1566 Dutch Holstein cows sampled from 18 herds in China, 22 herds across Denmark and 398 herds in the Netherlands. Stages of lactation of sampled cows ranged from 3 to 700 days in milk in the Chinese population, 9 to 481 days in milk in the Danish population and 60 to 278 days in milk in the Dutch Holstein cows. To standardize the samples from the three countries, only cows at days-in-milk of 60 and above were considered for the multi-population GWAS. Thus, 700 Chinese, 614 Danish and 1566 Dutch samples were available for the analysis. The reason to standardize the dataset by lactation stage is that the genetic determination of milk fat composition traits might be different in the early stages of lactation. There is evidence that effects of genes in early lactation differ from those later in lactation [65]. Excluding early lactation records can help eliminate this heterogeneity issue.

FA traits, including C8:0, C10:0, C12:0, C14:0, C14:1, C15:0, C16:0, C16:1, C18:0, C18:1c9, C18:2n6, C18:3n3 and C18:2 cis-9,trans-11 (CLA), were analyzed using the GC method. Details of the sample preparation for the GC analyses are as described by Li et al. [10] for Chinese samples, Poulsen et al. [66] for Danish samples and Stoop et al. [5] for Dutch samples. Genomic regions affecting the saturated FAs might show association to the unsaturated forms because the saturated form available for desaturation determines proportion of the unsaturated FAs. Hence, calculation of the desaturation indexes might allow detection of regions particularly associated with the desaturation process. Accordingly, desaturation indexes were calculated based on the FA measurements as: C14 index = C14:1/(C14:1 + C14:0) * 100; C16 index = C16:1/(C16:1 + C16:0) * 100 and C18 index = C18:1c9/ (C18:1c9 + C18:0) * 100.

### Genotypes and imputation

High-density (HD) genotypes, real or imputed, were available for all cows used in the analyses. All cows in the Chinese dataset were initially genotyped using the BovineSNP50 Beadchip (50 K, Illumina). A sample population of 96 Chinese Holstein bulls, genotyped using the BovineHD Beadchip (777 K), was available as reference to impute the 50 K genotypes of the cows to HD.

Part of the Danish cows were genotyped using the BovineSNP50 Beadchip, while the remaining Danish cows were genotyped using the BovineHD Beadchip and used as reference to impute the 50 K genotypes of the first part of the Danish cows to HD as described in Gebreyesus et al. [67]. The Dutch cows were genotyped with a custom 50 K SNP Beadchip and subsequently imputed to HD as presented in detail in Duchemin et al. [68]. SNPs with minor allele frequencies (MAF) less than 0.05 or with a count of one of the genotypes less than 10 in each population were excluded from the association analysis. A total of 464,130 SNPs were available for the association analysis. The SNP positions were based on the bovine genome assembly UMD 3.1 [69].

### Genome-wide association

A single-SNP association test was implemented using a mixed linear model in the GCTA program [70]. Association analysis was carried out using the following statistical model:

1 $$ {y}_{ijkl}=\mu + parit{y}_i+ her{d}_j+{b}_1\ast DI{M}_{ijkl}+{b}_2\ast SN{P}_k+ anima{l}_l+{e}_{ijkl}, $$

Where y_*ijkl*_ is the phenotype of cow *l*; μ is the fixed effect of mean; *parity*_*i*_ and *herd*_*j*_ are the fixed effects of parity and herd, respectively; b_*1*_ is the regression coefficient for DIM, DIM_*ijkl*_ is a covariate of days in milk; b_*2*_ is the allele substitution effect for SNP, SNP_*k*_ is a covariate indicating the number of copies of a specific allele (0, 1 or 2) of the SNP; and animal is the random additive genetic effect. Animal effects were assumed to be distributed as $\mathrm{N}\left(0,\mathbf{G}{\sigma}_a^2\right),$ where **G** is the genomic relationship matrix constructed using all HD genotypes but excluding the SNPs on the chromosome on which SNP *k* is located. Residuals were assumed to be distributed as: $\mathrm{N}\left(0,\mathbf{I}{\sigma}_e^2\right),$ where I is the identity matrix. Since only cows with more than 60 days-in-milk were included in the analyses, a linear adjustment for DIM was sufficient. For the FA traits C18:2n6, C18:3n3 and CLA, log transformation was applied prior to the association analysis to account for observed heterogeneity of residual variances.

Significance thresholds were determined using a false discovery rate (FDR). Significance thresholds corresponding to FDR of 5% ranged for different FA from –log10 *p*-value = 3.4 to –log10 *p*-value = 5.0. We used a –log10 *p*-value of 5.0 as the genome-wide significance threshold for all FA composition traits.

### Determining multiple regions on a chromosome

To determine if a region harbored one or more QTL, iterative approaches fitting the effect of SNPs with the highest –log 10 *p*-values were employed. In this approach, the SNP with the highest –log 10 *p*-value for the studied FA trait was considered as the lead SNP. The allelic dosage of such a lead SNP was then fitted as fixed effect for a second round of chromosome-wide analyses. If other SNPs, also significantly associated in the first round GWAS, were still found to have -log 10-pvalue > 5 in the second round analysis, the SNP with the highest –log 10 *p*-value in the second analysis was taken as the second lead SNP and its allelic dosage fitted as fixed effect for a third round of analysis. This procedure was iterated until no further SNP with -log 10-pvalue > 5 was observed. The SNPs that showed significant association in a round of GWAS but showed –log 10 *p*-value < 5 upon fitting the allelic dosage of the lead SNP were then considered as part of a region around that lead SNP. The position of the first and last such SNP before and after the lead SNP were considered as the boundaries of the region.

### Estimation of genetic variances explained by SNPs

Genetic variance explained by the lead SNP in a region was calculated from the GWAS summary as: *2pq*α^*2*^, where *p* and *q* are the allele frequencies and α is the allele substitution effect [71]. The proportion of total genetic variance explained by such a lead SNP was then calculated as:

$$ \raisebox{1ex}{$2 pq{\upalpha}^2$}\!\left/ \!\raisebox{-1ex}{${\sigma}_a^2$}\right., $$

where ${\sigma}_a^2$ is the additive genetic variance estimated using model 1 but without fitting fixed effects of SNP and using **G** constructed using all HD SNPs. Computation of genetic variance explained by SNPs from a GWAS summery might lead to overestimation of SNP effects [72] specially for small effect size SNPs that only just reach the significance threshold. Heritability (h^2^) estimates were computed as:

2 $$ {h}^2=\frac{\sigma_a^2}{\sigma_a^2+{\sigma}_e^2}, $$

where ${\sigma}_e^2$ is the residual variance.

### Gene assignment and enrichment analysis

Genes found within detected genomic regions were retrieved from the ensemble database using the BioMart web interface based on the UMD 3.1 bovine genome assembly (https://www.ensembl.org/biomart/martview). The DAVID functional annotation tool (https://david.ncifcrf.gov*)* was then used to analyze overrepresented GO biological terms, which included the terms cellular component (CC), molecular function (MF), biological process (BP) and the KEGG (Kyoto Encyclopedia of Genes and Genomes) pathways. Ontologies in the mammalian phenotype database were accessed and searched for genes connected to abnormalities relevant to FA metabolism using the Mouse Genome Informatics (MGI) web platform (http://www.informatics.jax.org/batch).

## Additional files

Additional file 1: Effects of the lead SNPs in all the detected genomic regions. SNP regression coefficients (SNP effects) in all the FA traits studied, along with the standard errors and –log 10 *p*-values, for the lead-SNP in each of the detected genomic region. (XLSX 45 kb)

Additional file 2: List of genes identified as enriched using the DAVID based enrichment analysis. Gene names and the positions in the genome (chromosome, start and end bp) clustered to at least one of the significantly enriched pathways and GO terms. (XLSX 24 kb)

Additional file 3: Micro-RNAs located within detected genomic regions. List of micro-RNAs (names and ensemble stable IDs) located in detected genomic regions and their positions in the genome (chromosome, start and end bp). (XLSX 11 kb)

## Abbreviations

BTA: *Bos Taurus* Autosome
FA: Fatty Acid
FDR: False Discovery Rate
GC: Gas Chromatography
GO: Gene Ontology
GWAS: Genome-Wide Association Study
HD: High-Density
IR: Infrared
KEGG: Kyoto Encyclopedia of Genes and Genomes
LD: Linkage Disequilibrium
PUFA: Poly-Unsaturated Fatty Acid
QTL: Quantitative Trait Loci
SNP: Single Nucleotide Polymorphism
